# Loss of heterozygosity on 10q and mutational status of *PTEN* and *BMPR1A* in colorectal primary tumours and metastases

**DOI:** 10.1038/sj.bjc.6601687

**Published:** 2004-03-02

**Authors:** M Karoui, C Tresallet, C Julie, U Zimmermann, F Staroz, A Brams, C Muti, C Boulard, A-M Robreau, H Puy, R Malafosse, C Penna, F-R Pruvot, J P Thiery, C Boileau, P Rougier, B Nordlinger, F Radvanyi, B Franc, H Hofmann-Radvanyi

**Affiliations:** 1 Fédération des Spécialités Digestives, Hôpital Ambroise Paré, AP-HP, Université Versailles-Saint Quentin en Yvelines, 92104 Boulogne Cedex, France; 2 Service d'Anatomie et de Cytologie Pathologiques, Hôpital Ambroise Paré, AP-HP, Université Versailles-Saint Quentin en Yvelines, 92104 Boulogne Cedex, France; 3 Service de Génétique, Hôpital Ambroise Paré, AP-HP, 92104 Boulogne Cedex, France; 4 UMR 144, CNRS-Institut Curie, 75248 Paris Cedex 05, France; 5 Laboratoire de Biochimie, Hôpital Louis Mourier, AP-HP, 92701 Colombes Cedex, France; 6 Service de Chirurgie Digestive et de Transplantation, Hôpital Claude Huriez, CHRU de Lille, 59037 Lille Cedex, France; 7 Laboratoire de Biochimie et de Génétique Moléculaire, Hôpital Ambroise Paré, AP-HP, Université Versailles-Saint Quentin en Yvelines, 9 Avenue Charles de Gaulle, 92104 Boulogne Cedex, France; 8 Service de Chirurgie Digestive, Hôpital Pitié-Salpêtrière, AP-HP, 75651 Paris Cedex 13, France

**Keywords:** chromosome 10, LOH, colorectal cancer, metastasis, PTEN, BMPR1A

## Abstract

We investigated the possible role of chromosome 10q losses in colorectal cancer metastasis by carrying out an allelic imbalance study on a series of microsatellite instability-negative (MSI−) primary tumours (*n*=32) and metastases (*n*=36) from 49 patients. Our results demonstrate that 10q allelic losses are associated with a significant proportion (25%) of MSI− colorectal tumours, but are not involved in the metastatic process. *PTEN* and *BMPR1A*, two genes located in the common deleted region, were screened for mutations in samples with loss of heterozygosity. The absence or low frequency of mutations indicates that the inactivation of these genes by deletion of one allele and mutation of the other one plays only a minor role in MSI− tumours.

Colorectal carcinoma is one of the most common cancers in Western countries. Most deaths related to colorectal cancer are caused by metastasis. Little is known about the genetic alterations associated with the metastatic phenotype. Deletions of the long arm of chromosome 10 have been reported in many types of tumour, including colorectal carcinomas (Frayling *et al*, 1997), and are correlated with tumour progression and/or metastasis formation in several of these cancers, such as glial tumours (Balesaria *et al*, 1999), lung cancer (Petersen *et al*, 1998), head and neck squamous cell carcinomas (Bockmuhl *et al*, 2002), bladder (Cappellen *et al*, 1997), prostate (Komiya *et al*, 1996) and breast carcinomas (Bose *et al*, 1998). Several putative or known tumour-suppressor genes have been mapped to 10q, including *BMPR1A* on 10q23.2 and *PTEN/MMAC1/TEP1* on 10q23.3. Mutations in *PTEN* are associated with hereditary cancer predisposition syndromes (Liaw *et al*, 1997; Marsh *et al*, 1997) and, to a greater or lesser extent, with a wide variety of sporadic cancers (Ali *et al*, 1999; Bonneau and Longy, 2000). With the exception of endometrial cancer (Mutter *et al*, 2000), alterations to *PTEN* in cancer are almost exclusively detected in advanced stages of disease. Mutations in *PTEN* have been studied only in primary colorectal tumours, and this gene appears to be involved only in tumours with microsatellite instability (MSI+) (Guanti *et al*, 2000; Shin *et al*, 2001; Zhou *et al*, 2002). The presence of germ-line-inactivating mutations in the *BMPR1A* gene has been found to be responsible for a significant proportion of cases of juvenile polyposis syndrome, an inherited hamartomatous polyposis syndrome with a risk of colon cancer (Howe *et al*, 2001; Zhou *et al*, 2001). Although *BMPR1A* was a good candidate for involvement in the pathogenesis of sporadic colon cancer, no mutations have yet been identified in primary colorectal tumours displaying LOH at the BMPR1A locus (Howe *et al*, 2001).

As losses on chromosome 10q have frequently been associated with tumour progression, we carried out an allelic imbalance study on a series of MSI− colorectal tumour samples consisting of 32 primary tumours at various stages and 36 distant metastases. In 19 cases, metastases and primary tumours were obtained from the same patient. The involvement of two candidate genes located in the minimal region of allelic deletion, *PTEN* and *BMPR1A*, was assessed by mutational analysis.

## MATERIALS AND METHODS

### Patients and tissue samples

The primary colorectal carcinomas and metastases were obtained from patients who underwent surgery at Ambroise Paré Hospital (Boulogne, France). In all cases, ethical approval and appropriate consent were obtained. Detailed information on the clinical and histological features is provided in Appendix A.

### DNA extraction

Frozen or formalin-fixed paraffin-embedded tissues were serially sectioned onto slides and tumour tissue was microdissected. DNA was then extracted as described by Billerey *et al* (2001). Constitutional DNA for each patient was obtained from blood leukocytes, or from normal tissues (uninvolved colon mucosa or liver) in the surgical specimens.

### RNA extraction and reverse transcription

Total RNA was isolated from frozen tissues, using the guanidine isothiocyanate/caesium chloride cushion method, and was used as a template for first-strand cDNA synthesis by random priming, as previously described (Diez de Medina *et al*, 1997).

### Analysis of 10q microsatellite loci

Tumours with high microsatellite instability (H-MSI) (Boland *et al*, 1998) were excluded from the study. Allelic imbalance was evaluated at 32 loci distributed along chromosome 10q. PCR products were subjected to electrophoresis in a 6% acrylamide sequencing gel under denaturing conditions. DNA was transferred onto Hybond N+ membranes (Amersham, Little Chalfont, UK). PCR products were detected using a DIG 3′ end-labelled specific oligonucleotide primer or a (CA)_14_ repeat probe. For normal and tumour tissue pairs for which allelic imbalance or retention of heterozygosity was not clear, membranes were reprobed with a ^32^P end-labelled probe. Signals were then quantified with a Storm 840 PhosphorImager (Molecular Dynamics, Sunnyvale, CA, USA). In informative cases, allelic imbalance was considered to be present if a difference of at least 40% was observed in allelic ratios between tumoural and normal DNA from a given patient.

### Mutational analysis by SSCP and heteroduplex analysis

SSCP (Single-Strand Confirmation Polymorphism) was used for cDNA analysis, with overlapping primer pairs covering the entire coding region of *PTEN* or *BMPR1A*. Heteroduplex analysis was performed as a complementary mutation-screening method for genomic DNA, using primer pairs covering all coding exons, exon–intron junctions, and more than 50 bp of flanking intronic sequences. The sequences of the primers used are available on request.

### Sequence analysis

Electrophoresis variants predicted by SSCP or heteroduplex analysis were confirmed by direct sequencing, using the ABI Prism Dye Terminator Sequencing Ready Reaction Kit (*PE* Biosystems, Courtaboeuf, France), according to the manufacturer's instructions.

### Statistical analysis

Two-tailed Fisher's exact tests were used for statistical analyses. Differences were considered significant if the two-tailed *P*-value was <0.05.

## RESULTS

### Identification of the region of allelic loss on chromosome 10q

**Figure 1:**
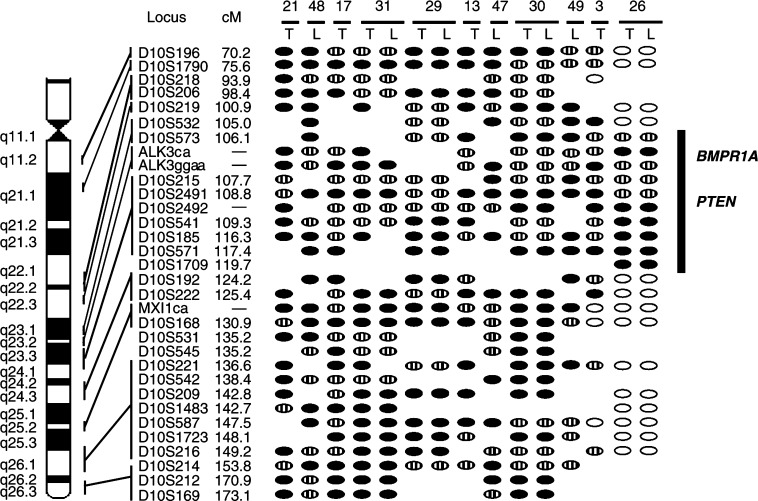
Deletion mapping of chromosome 10q. Allelic patterns of chromosome 10q for all tumour samples with LOH are shown. T: primary tumour; L: liver metastasis. Plain ovals: no loss of heterozygosity in the tumour sample; black ovals: loss of heterozygosity in the tumour sample; striped ovals: not informative (homozygosity in the normal sample); blank space: not done. Names of microsatellite markers studied, their positions on 10q and their genetic distance to the top of the chromosome are indicated on the left. The minimal region of loss and the location of the *BMPR1A* and *PTEN* genes are shown on the right.

In all, 11 out of 49 patients (22.4%) presented losses on 10q (Figure 1

). Out of the 15 tumour specimens (eight primary tumours and seven metastases) with loss of heterozygosity (LOH) from these 11 patients, 12 displayed losses of all or most of chromosome 10q. The three remaining samples displayed similar partial losses of chromosome 10q. The tumour and the metastasis from case #26 defined a minimal region of allelic deletion flanked proximally by D10S532, and distally by D10S192. This 19-centimorgan minimal region corresponds to the cytogenetic location 10q23–q24 and includes the two tumour-suppressor genes *PTEN* and *BMPR1A*.

### Allelic losses in primary tumours and distant metastases

Two of the 13 colorectal carcinomas that did not develop metastases more than 5 years after primary tumour resection (cases #3 and 13), six of the 19 primary tumours that did develop synchronous or metachronous metastases (cases #17, 21, 26, 29, 30 and 31) and seven of the 36 metastases analysed (cases #26, 29, 30, 31, 47, 48 and 49) displayed chromosome 10q losses (Figure 1). The percentages of chromosome 10q loss did not differ significantly in these three groups (*P*>0.3).

Loss of heterozygosity analysis in the 19 pairs of primary colorectal carcinomas and corresponding metastases available revealed losses in six cases (cases #17, 21, 26, 29, 30 and 31). Concordant patterns of loss were observed in four pairs (cases #26, 29, 30 and 31). Two pairs (cases #17 and 21) showed LOH in the primary tumour, and retention of heterozygosity in the metastatic tumour. No losses were seen in the metastasis only (Table 1

**Table 1:** LOH in primary tumour and corresponding metastasis pairs

**Case**	**LOH in the primary tumour**	**LOH in the corresponding metastasis**
26	+	+
29	+	+
30	+	+
31	+	+
17	+	−
21	+	−

).

### Mutation screening of *PTEN* and *BMPR1A*

**Figure 2:**
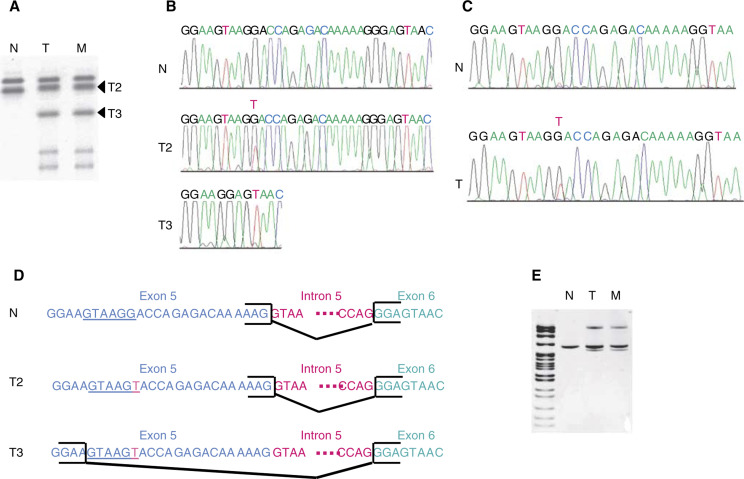
PTEN mutation in the primary tumour and liver metastasis of case 26. (**A**) Abnormal bands were detected by SSCP analysis of cDNA from the primary tumour (T) and liver metastasis (M) using primers in exon 5 (sense) and exon 6 (antisense). These bands were not present in normal colon cDNA from the same patient (N). (**B**) Sequencing analysis of the two main abnormal bands (T2) and (T3) present in the primary tumour. Sequence of the normal cDNA from the same patient (N). (**C**) Sequencing of the genomic DNA of the primary tumour (T) and corresponding normal tissue (N). Tumour DNA harboured a G to T point mutation. (**D**) The various alternatively spliced forms deduced from the cDNA and genomic sequences presented in (**B**) and (**C**) are shown. The T2 allele carrying a G/T transversion in exon 5 presented the same splice form as the normal allele. T3 showed a 21 bp deletion at the 3′ end of exon 5. The new consensus donor splice site created by the mutation is underlined. (**E**) RT–PCR analysis of the primary tumour (T), liver metastasis (M) and corresponding normal tissue using the same primers as in (**A**). Lane 1: pBR322 DNA-*MSPI* digest.

Samples with LOH on 10q were analysed for *PTEN* and *BMPR1A* mutations at DNA and transcript levels. Several extra bands were detected by SSCP analysis of exon 5 in the cDNA of the primary tumour and liver metastasis of case #26 (Figure 2A

). Sequencing of one of these abnormal bands (T2) revealed a G → T transversion in exon 5, and sequencing of another such band (T3) revealed a 21 bp deletion of the 3′ end of exon 5 (Figure 2B). Analysis of normal tissue from the patient showed only the normal sequence, demonstrating that these variants occurred somatically. The sequencing of genomic DNA showed that the primary tumour and metastasis of this case harboured the G to T point mutation in exon 5 (Figure 2C). This mutation, at the third base of codon 159, is expected to cause an arginine-to-serine substitution in the tyrosine phosphatase domain, and creates a new donor-splice site (GTAAGG → GTAAGT). Several aberrant transcripts were generated by alternative splicing involving this new donor site, as shown in Figure 2D and E. None of the samples investigated by SSCP analysis or HDA showed evidence of *BMPR1A* mutations.

## DISCUSSION

Of the 49 cases included in this study (22.4%), 11 presented allelic losses on 10q, indicating that structural alterations of chromosome 10q occur relatively frequently in colorectal carcinogenesis. The percentage of 10q loss did not differ significantly between the group of primary tumours without metastasis within 5 years, the group of primary tumours that did develop synchronous or metachronous metastasis and the group of distant metastases. Although the number of primary tumours without metastasis at 5 years in our study was small, our findings suggest that LOH on chromosome 10q is probably not an important event in metastasis formation. This hypothesis is supported by the finding that two primary tumours exhibited chromosome 10 losses with no deletion in the corresponding metastases, and that no losses were observed in metastases alone. Our results also suggest that chromosome 10q loss is a relatively late event in the history of the primary tumour.

The 19 cM minimal region of deletion defined here is included within the very large region (10p13–10q24) previously reported by Frayling *et al* (1997). It contains two suppressor genes, *PTEN* and *BMPR1A*. The frequency of LOH (22.4%) that we found at these loci was similar to those (18–24%) reported in previous studies (Howe *et al*, 2001; Zhou *et al*, 2002).

We identified no *BMPR1A* mutations in tumour samples showing LOH on chromosome 10q. One single *PTEN* mutation was found, located in exon 5, a hotspot for mutation. This mutation, described here for the first time, has two consequences: it leads to the replacement of a highly conserved residue in the phosphatase domain and generates a new donor splice site. The identification of only one tumour with a *PTEN* mutation in our series of MSI− tumours, consistent with the recent results of Zhou *et al* (2002), indicates that the inactivation of *PTEN* by mutation is a rare event in MSI− colorectal tumours and is essentially restricted to the MSI+ pathway (Guanti *et al*, 2000; Shin *et al*, 2001; Zhou *et al*, 2002).

Metastasis is the major complication in cancer progression. Very few studies have examined chromosomal alterations in colorectal metastases. We show here that neither losses on chromosome 10q nor *PTEN* and *BMPR1A* mutations seem to play a role in the metastasic process.

## Appendix A

Detailed information on the clinical and histological features is summarised in Table A1

**Table A1:** Clinical and pathological features

**Case**	**Sex**	**Age (years)**	**Primary tumour site**	**TNM stage**	**Metastasis site**	**Synchronous/metachronous^a^**
1	F	63	Right colon	T1N0M0		
2	M	83	Right colon	T3N0M0		
3	F	51	Transverse colon	T3N0M0		
4	F	57	Sigmoid colon	T3N0M0		
5	M	72	Sigmoid colon	T3N0M0		
6	M	61	Sigmoid colon	T3N0M0		
7	F	61	Right colon	T3N1M0		
8	F	54	Right colon	T3N1M0		
9	M	61	Descending colon	T3N1M0		
10	F	54	Rectum	T3N1M0		
11	F	46	Rectum	T3N2M0		
12	M	83	Right colon	T3N2M0		
13	F	39	Rectum	T3N2M0		
14	M	66	Sigmoid colon	T1N0M0	Liver	M
15	M	78	Rectum	T2N0M0	Liver	M
16	F	78	Right colon	T3N0M0	Liver	M
17	F	66	Rectum	T3N2M0	Liver	M
18	F	44	Right colon	T3N0M1	Liver	S
19	F	68	Sigmoid colon	T3N0M1	Liver	S
20	M	63	Sigmoid colon	T3N0M1	Liver	S
21	F	46	Right colon	T3N0M1	Ovary	S
22	M	72	Sigmoid colon	T3N0M1	Peritoneum	S
23	F	70	Sigmoid colon	T3N0M1	Peritoneum	S
24	F	59	Rectum	T2N1M1	Liver	S
25	M	71	Right colon	T3N1M1	Liver	S
26	M	66	Sigmoid colon	T3N1M1	Liver	S
27	M	62	Descending colon	T3N2M1	Liver	S
28	M	78	Sigmoid colon	T3N2M1	Liver	S
29	F	79	Sigmoid colon	T3N2M1	Liver	S
30	M	64	Rectum	T3N2M1	Liver	S
31	F	61	Transverse colon	T4N2M1	Liver	S
32	F	39	Rectum	T4N2M1	Liver	S
33	F	73	Right colon	TisN0M0	Liver	M
34	M	39	Sigmoid colon	T2N0M0	Liver	M
35	F	53	Sigmoid colon	T2N1M0	Liver	M
36	F	69	Sigmoid colon	T3N0M0	Liver	M
37	M	48	Sigmoid colon	T3N0M0	Liver	M
38	M	70	Descending colon	T3N1M0	Liver	M
39	M	61	Descending colon	T3N1M0	Liver	M
40	F	54	Sigmoid colon	T3N1M0	Liver	M
41	M	61	Right colon	T3N1M0	Liver	M
42	M	57	Sigmoid colon	T3N1M0	Liver	M
43	M	61	Right colon	T3N1M0	Liver	M
44	F	55	Rectum	T3N2M0	Liver	M
45	F	69	Sigmoid colon	T2N0M1	Liver	S
46	M	51	Sigmoid colon	T2N1M1	Liver	S
47	F	74	Rectum	T3N1M1	Liver	S
48	M	60	Sigmoid colon	T3N1M1	Liver	S
49	F	76	Sigmoid colon	T3N1M1	Liver	S

The patients for whom no metastasis is indicated (cases 1–13) did not develop metastasis during the 5 or more years following the resection of the primary tumour.

a Synchronous metastases are those detected at the time of primary tumour diagnosis.

Primary tumours from cases 33 to 49 were not available for this study.

.
